# Pulmonary Perfusion and Ventilation during Cardiopulmonary Bypass Are Not Associated with Improved Postoperative Outcomes after Cardiac Surgery

**DOI:** 10.3389/fcvm.2016.00047

**Published:** 2016-11-28

**Authors:** Yiliam F. Rodriguez-Blanco, Angela Gologorsky, Tomas Antonio Salerno, Kaming Lo, Edward Gologorsky

**Affiliations:** ^1^Anesthesiology, University of Miami Miller School of Medicine, Miami, FL, USA; ^2^Private Practice, Anesthesiology, Miami, FL, USA; ^3^Cardiovascular Surgery, University of Miami Miller School of Medicine, Miami, FL, USA; ^4^Division of Biostatistics, Department of Public Health Sciences, University of Miami, Miami, FL, USA; ^5^Anesthesiology, Allegheny General Hospital, Pittsburgh, PA, USA

**Keywords:** cardiopulmonary bypass, postoperative complications, pulmonary perfusion, pulmonary ventilation, cardiac surgery outcome

## Abstract

**Objectives:**

Clinical trials of either pulmonary perfusion or ventilation during cardiopulmonary bypass (CBP) are equivocal. We hypothesized that to achieve significant improvement in outcomes both interventions had to be concurrent.

**Design:**

Retrospective case–control study.

**Settings:**

Major academic tertiary referral medical center.

**Participants:**

Two hundred seventy-four consecutive patients who underwent open heart surgery with CBP 2009–2013.

**Interventions:**

The outcomes of 86 patients who received pulmonary perfusion and ventilation during CBP were retrospectively compared to the control group of 188 patients.

**Measurements and main results:**

Respiratory complications rates were similar in both groups (33.7 vs. 33.5%), as were the rates of postoperative pneumonia (4.7 vs. 4.3%), pleural effusions (13.9 vs. 12.2%), and re-intubations (9.3 vs. 9.1%). Rates of adverse postoperative cardiac events including ventricular tachycardia (9.3 vs. 8.5%) and atrial fibrillation (33.7 vs. 28.2%) were equivalent in both groups. Incidence of sepsis (8.1 vs. 5.3%), postoperative stroke (2.3 vs. 2.1%), acute kidney injury (2.3 vs. 3.7%), and renal failure (5.8 vs. 3.7%) was likewise comparable. Despite similar transfusion requirements, coagulopathy (12.8 vs. 5.3%, *p* = 0.031) and the need for mediastinal re-exploration (17.4 vs. 9.6%, *p* = 0.0633) were observed more frequently in the pulmonary perfusion and ventilation group, but the difference did not reach the statistical significance. Intensive care unit (ICU) and hospital stays, and the ICU readmission rates (7.0 vs. 8.0%) were similar in both groups.

**Conclusion:**

Simultaneous pulmonary perfusion and ventilation during CBP were not associated with improved clinical outcomes.

## Introduction

Pulmonary dysfunction after open heart surgery is multifactorial. The relative contributions of various pathophysiologic factors remain obscure despite enormous experimental and clinical efforts (1–3). Cessation of alveolar ventilation and perfusion engendered in traditional technique of cardiopulmonary bypass (CPB) are thought to potentiate the effects of systemic inflammatory reaction associated with CPB and to contribute to postoperative pulmonary dysfunction. However, despite initial enthusiasm, attempts to mitigate iatrogenic atelectasis and alveolar ischemia had very limited impact on postoperative pulmonary dysfunction (4–7). We have argued in the past (8) that the disappointing inconsistency of these clinical trials stemmed from their focus on either pulmonary perfusion or ventilation but never on both simultaneously; we had proposed that binary interventions of either ventilation of ischemic alveoli or perfusion of atelectatic lungs were of unlikely benefit, and that to achieve favorable outcomes both interventions had to be concurrent; lungs had to be both perfused and ventilated during CPB (9). Therefore, we have studied the effects of simultaneous pulmonary perfusion and ventilation during CPB in comparison to traditional management of CPB. We hypothesized that the pulmonary perfusion and ventilation was a safe technique that had the potential to reduce the incidence and severity of postoperative pulmonary dysfunction in patients undergoing open heart surgery.

## Materials and Methods

Following Institutional Review Board (IRB) approval, the medical records of 274 consecutive patients who underwent a cardiac valve procedure at Jackson Memorial Hospital/University of Miami between January 1, 2009 and December 30, 2013 were reviewed. No specific criteria were implemented other than cardiac surgery requiring CBP during the designated time frame. All data were anonymized before analysis. The informed consent for this retrospective study was waived.

### Data Collection

All perioperative data were obtained from the electronic medical records. The collected data included multiple descriptors of patients’ demographics, comorbidities, details of intraoperative course, perioperative transfusion requirements, duration of the intensive care unit (ICU) and hospital stays, and perioperative mortality and morbidity events. Postoperative pulmonary complications, re-intubation rates, need for postoperative re-exploration and transfusion, and adverse neurologic and renal events were all included in the analysis.

### Outcomes

The outcomes of 86 patients who received pulmonary perfusion and ventilation during CPB (cohort of interest) were retrospectively compared to the outcomes of control group of 188 patients who had undergone conventional CPB management without pulmonary perfusion and ventilation.

The primary outcomes included the in-hospital morbidity and mortality rates. Secondary outcome measures included various postoperative complications including cardiac, pulmonary, neurologic and renal compromise, sepsis, duration of postoperative mechanical ventilator support in the ICU, length of ICU and hospital stays, and need for re-exploration and transfusion requirements.

### Intraoperative Management of Cardiopulmonary Bypass

As a matter of surgical preference, in all patients, every attempt was made to avoid or to minimize the duration of cardioplegic arrest utilizing the “beating heart technique” (10, 11). While the systemic circulation was supported by CPB, the unloaded (“empty”) beating heart was continuously perfused (both in antegrade and retrograde fashion) with warm oxygenated blood. In certain circumstances, such as a difficult valve exposure, an excessively bloody field, or a perceived increased risk of embolization, cardioplegic arrest was induced. All the decisions regarding the administration of hyperkalemic cardioplegia and the duration of cardioplegic arrest were made by the surgeon based on the technical aspects of the surgery.

In the control group, pulmonary ventilation was suspended for the duration of the CPB, and no attempt was made to perfuse the pulmonary artery. In the cohort of interest, the following technique was used: pulmonary ventilation was maintained with inspired oxygen fraction (FiO_2_) of 0.5, tidal volumes of 4–6 ml/kg of ideal weight, at a rate of 5 breaths/min, and end-expiratory pressure (PEEP) of 5 mmH_2_O. The pulmonary artery was perfused *via* a 3-mm diameter perfusion cannula attached to the aortic cannula. In this setup, pulmonary artery perfusion depended on the CPB flow and pressure, tubing length and resistance, and blood viscosity. At systemic flows of 2.4 l/min/m^2^ and mean arterial pressures of 60 mmHg, Doppler-measured average pulmonary arterial flows ranged between 400 and 500 ml/min. Presence of the alveolar perfusion was confirmed by steady values of end-tidal CO_2_ on continuous capnography (12). The decision to utilize the pulmonary perfusion and ventilation during CPB was exclusively the surgeon’s.

### Statistical Analysis

Descriptive statistics were used to describe all variables of interest within each of the comparison groups (lung perfusion/ventilation group vs. conventional technique group). Mean values and SDs were calculated for continuous variables that approximately follow the normal distribution and Student’s *t*-tests were then used to compare the groups. Median values and interquartile ranges (IQR) were calculated for variables that were highly skewed and Wilcoxon’s rank-sum tests were then used for comparison between the groups. Frequencies and proportions were used to analyze categorical variables. Chi-square tests or Fisher’s exact tests were then used depending on the expected cell counts, to compare difference in proportions between the groups and to examine the overall trends. Due to the amount of comparisons performed, a Bonferroni adjusted type I error rate of 0.00091 was assumed for significance. All analyses were performed using SAS 9.4 (SAS Institute, Cary, NC, USA).

## Results

Preoperative demographics and clinical data are summarized in Table 1. Baseline demographics were similar between the groups. Of the 86 patients who received pulmonary perfusion and ventilation during CPB, most were male (59.3%) with a mean age of 58 years and a median left ventricular ejection fraction (EF) of 55%. Comparably, of 188 patients in the control group, most were male (53.2%), with a mean age of 57 and EF of 55%. The most common comorbidities in both groups included hypertension (67.4 vs. 78.7%), dyslipidemia (34.9 vs. 39.3%), smoking (35 vs. 30%), diabetes (30 vs. 27%), chronic lung disease (22 vs. 19%), and renal disease (19 vs. 10%). Even though the patients in the cohort of interest had higher incidence of a prior cerebrovascular event (19.7 vs. 10.1%), coronary artery disease (12.8 vs. 5.3%), prior coronary artery bypass grafting (CABG) (4.7 vs. 2.1%), prior valve surgery (17.4 vs. 7.9%), and preoperative ICU admissions (36.1 vs. 20.7%), the difference was not statistically significant when Bonferroni correction to the *p* values was applied. Patients in both groups had comparable rates of intubation and mechanical ventilation immediately prior to the surgery (3.5 vs. 5.3%).

**Table 1:** **Demographic data**.

Demographic data	Lung V/Q group (*n* = 86)			Conventional (*n* = 188)			Stats	
	Frequency		%	Frequency		%	*p*-Value	Test
Gender								
Female	35		40.7	88		46.8	0.3453	
Male	51		59.3	100		53.2		
Smoker	30		34.9	57		30.3	0.4513	c
Diabetes	26		30.2	51		27.1	0.5957	c
HTN	58		67.4	148		78.7	0.0448	c
Dyslipidemia	30		34.9	74		39.4	0.4784	c
PVD	5		5.8	3		1.6	0.1134	f
CVA	17		19.8	19		10.1	0.028	c
Chronic lung disease	19		22.1	35		18.6	0.5021	c
History of renal failure	17		19.8	19		10.1	0.028	c
History of dialysis	10		11.6	8		4.3	0.0223	c
Prior CABG	4		4.6	4		2.1	0.2496	f
Prior valve surgery	15		17.4	15		8.0	0.0199	c
History of prior CAD	11		12.8	10		5.3	0.031	c
Intubated before surgery	3		3.5	10		5.3	0.7605	f
ICU admission before surgery day	31		36.1	39		20.7	0.007	c

	***N***	**Median**	**IQR**	***N***	**Median**	**IQR**	***p*-Value**	**Test**

Ejection fraction	82	55	20	179	55	10	0.8858	

	***N***	**Mean**	**SD**	***N***	**Mean**	**SD**	***p*-Value**	**Test**

Age	86	58.6	15.2	188	57.7	14.1	0.6501	

*OR, operating room; HTN, hypertension; CVA, cerebrovascular accident; PVD, peripheral vascular disease; CAD, coronary artery disease; CABG, coronary artery bypass graft; ICU, intensive care unit; V/Q, pulmonary ventilation and perfusion*.

*Test: c, chi-square test; f, Fischer’s exact test, *p*-value (adjusted alpha 0.0009)*.

Table 2 specifies the types of surgical procedures and summarizes the intraoperative data. In both groups, single valve surgery was the most common (77.9% in the cohort of interest vs. 80.9% in the control group); rates of multiple valves surgery (10.5 vs. 7.5%) and of combined valve and CABG procedures (11.6 vs. 11.7%) were similar in both groups (*p* = 0.703). Transfusion rates (calculated as fraction of patients requiring at least 1 U of autologous blood, %) (94.2 vs. 95.7%) and the average duration of surgery (time from the surgical incision to the end of surgery) were equal in both groups (4.5 vs. 4.7 h).

**Table 2:** **Intraoperative data**.

Intraoperative data	Lung V/Q group (*n* = 86)		Conventional (*n* = 188)		Stats
	Frequency	%	Frequency	%	*p*-Value
Scheduled procedure					0.703
Single valve	67	77.9	152	80.8	
Double/triple valve	9	10.5	14	7.4	
CABG plus valve	10	11.6	22	11.7	
Intra-op blood products	81	94.2	180	95.7	0.5538^a^
Extubation in the OR	32	37.2	26	13.8	<0.0001

	**Median**	**IQR**	**Median**	**IQR**	***p*-Value**

CPB time, min	88	41	119	75	<0.0001
Time from incision to end of surgery, h	4.5	1.9	4.725	2.5	0.0515
Time from end of surgery to out of OR, min	25	22	25	20	0.3748

*OR, operating room; CABG, coronary artery bypass graft; V/Q, pulmonary ventilation and perfusion; CPB, cardiopulmonary bypass; min, minutes*.

*p-Value (adjusted alpha 0.0009). All associations involving categorical variables were examined using chi-square tests or Fisher exact tests (^a^)*.

Patients who had pulmonary perfusion and ventilation during CPB had significantly shorter CPB times (median 88 vs. 119 min, *p* < 0.0001) and higher rates of extubation in the OR (37.2 vs. 13.8%, *p* < 0001).

Table 3 summarizes some postoperative events. Overall, patients in both groups had similar transfusion requirements (calculated as the fraction of patients requiring at least 1 U of autologous blood, %) (60.5 vs. 50.5%, *p* = 0.126), duration of ICU (5 vs. 5 days) and hospital stays (9.3 vs. 9.5 days), and the ICU readmission rates (7.0 vs. 8.0%).

**Table 3:** **Postoperative course**.

Postoperative course	Pulmonary V/Q group (*n* = 86)		Conventional (*n* = 188)		Stats
	Median	IQR	Median	IQR	*p*-Value
ICU stay, days	5	5	5	5	0.6742
Postoperative hospital stay, days	9.3	12	9.5	7.4	0.3209

	Frequency	%	Frequency	%	*p*-Value

Postoperative blood products transfusion	52	60.5	95	50.5	0.126
ICU readmissions	6	6.9	15	7.9	0.7723

*ICU, intensive care unit; V/Q, ventilation and perfusion*.

*p-Value (adjusted alpha 0.0009). All associations involving categorical variables were examined using Chi-square tests or Fisher exact tests*.

Postoperative complications are tabulated in Table 4. Overall respiratory complications rates were similar in both groups (33.7 vs. 33.5%), as were the rates of postoperative pneumonia (4.7 vs. 4.3%), pleural effusions (13.9 vs. 12.2%), and re-intubations due to deterioration of pulmonary function (9.3 vs. 9.1%). Rates of adverse postoperative cardiac events, such as ventricular tachycardia (9.3 vs. 8.5%), atrial fibrillation (33.7 vs. 28.2%), and cardiac arrest (11.6 vs. 10.6%), were equivalent in both groups. Incidence of sepsis (8.1 vs. 5.3%), new postoperative stroke (2.3 vs. 2.1%), acute kidney injury (2.3 vs. 3.7%), and renal failure (5.8 vs. 3.7%) were similar in both groups.

**Table 4:** **Postoperative morbidity and mortality**.

Morbidity and mortality events	Lung V/Q group (*n* = 86)		Conventional (*n* = 188)		Stats	
	Frequency	%	Frequency	%	*p*-Value	Test
Respiratory	29	33.7	63	33.5	0.9727	c
Atelectasis	20	23.3	37	19.7	0.4987	c
Pneumothorax	4	4.6	13	6.9	0.471	c
Pleural effusion	12	13.9	23	12.2	0.6923	c
Pneumonia	4	4.6	8	4.3	1	f
Pulmonary embolism	0	0	1	0.5	–	–
Septic	7	8.1	10	5.3	0.3691	c
Stroke	2	2.3	4	2.1	1	f
AKI	2	2.3	7	3.7	0.7244	f
ARF	5	5.8	7	3.7	0.526	f
Cardiac arrest	10	11.6	20	10.6	0.8077	c
Coagulopathy	11	12.8	10	5.3	0.031	c
Cardiac tamponade	2	2.3	9	4.8	0.5112	f
Atrial fibrillation	29	33.7	53	28.2	0.3537	c
Ventricular arrhythmias	8	9.3	16	8.5	0.8297	c
Re-exploration	15	17.4	18	9.6	0.0633	c
Re-intubation	8	9.3	17	9.0	0.9448	c
Overall mortality	14	16.3	16	8.5	0.056	c

*V/Q, ventilation and perfusion; AKI, acute kidney injury; ARF, acute renal failure*.

*Test: c, chi-square test; f, Fischer’s exact test*.

Coagulopathy (12.8 vs. 5.3%, *p* = 0.031) and the need for mediastinal re-exploration (17.4 vs. 9.6%, *p* = 0.0633) were observed more frequently in pulmonary perfusion and ventilation group, but the difference did not reach the statistical significance.

Table 5 lists the mortality events for both groups. Cardiac failure was the leading cause of mortality in patients after pulmonary perfusion and ventilation during CPB (8 out of 14 patients). Multisystem organ failure [defined as systemic inflammatory response syndrome (SIRS) with or without documented systemic infection] contributed to mortality in four patients, and respiratory failure (defined as persistent, severe, and refractory hypoxemia) was seen in three patients. In the control group, 8 out of 16 patients succumbed to septic complications and SIRS with multisystem organ failure, 8 to postoperative cardiac failure, and 4 to severe respiratory failure. Overall mortality rates, though higher in the cohort of interest, were not statistically different (16.3 vs. 8.5%, *p* = 0.056).

**Table 5:** **Mortality events**.

Pulmonary perfusion/ventilation group		Conventional technique	

Procedure	Events	Procedure	Events
MV replacement (endocarditis)	Septic shock and multisystem organ failure	MV and TV repair + AVR	Septic shock and multisystem organ failure

AVR	Cardiogenic shock due to postoperative MI	MV replacement	Sternal wound dehiscence, mediastinitis, sepsis, multisystem organ failure

Redo MV replacement (preoperative endocarditis)	MRSA bacteremia, septic shock, ARDS	Bentall procedure	Cardiac arrest

Emergency MV replacement due to preoperative MI complicated with PM rupture; ECMO	Cardiogenic shock	AVR	Septic shock and multisystem organ failure

Emergency AVR due to severe acute AR and pulmonary edema after attempted valvuloplasty	Cardiogenic shock	AVR CABGx2	Severe postoperative respiratory failure required ECMO

MV replacement	Severe paravalvular leak, complicated by ARDS	AVR	Post-op cardiogenic shock; SIRS, multisystem organ failure

MV repair	Exacerbation of CHF and ARF due to rejection of transplanted kidney, cardiogenic shock	AVR CABGx1	Severe postoperative respiratory failure

AVR	Massive stroke with irreversible brain injury	Emergency MV replacement due to endocarditis and stroke	Severe postoperative respiratory failure requiring ECMO support, septic shock, multisystem organ failure

Emergency AVR CABGx2 due to left main coronary occlusion and AS	Cardiogenic shock, SIRS complicated with multisystem organ failure	AVR CABGx1	Refractory cardiogenic shock

Emergency MV replacement CABGx1 due to MI and PM rupture	Cardiogenic shock; SIRS complicated with multisystem organ failure	AVR, MV replacement due to endocarditis following major burn injury	ARDS, SIRS, multisystem organ failure

MV replacement	Respiratory failure	AVR CABGx1	Developed delayed symptomatic lung infiltrates requiring re-intubation; succumbed later to refractory respiratory failure

MV replacement	Cardiogenic shock	MV replacement due to endocarditis	Sepsis, multisystem organ failure

Emergency TV repair due to endocarditis	Postoperative septic shock complicated with severe heart failure, required postoperative ECMO support	Bentall procedure	Postoperative sternal wound infection, sepsis, multisystem organ failure

Redo (×3) MV replacement, TV repair due to endocarditis	SIRS, multisystem organ failure	AVR	Cardiogenic shock, unable to wean off CPB, postoperative ECMO
		AVR	Cardiac arrest in the ICU
		AVR due to endocarditis	Cardiogenic shock

*MV, mitral valve; AVR, aortic valve replacement; TV, tricuspid valve; CABGx1, coronary artery bypass grafting, CABGx2, coronary artery bypass grafting, two vessels; one vessel; SIRS, systemic inflammatory response syndrome; MRSA, methicillin-resistant Staphylococcus aureus; ARDS, acute respiratory distress syndrome; CPB, cardiopulmonary bypass; ECMO, extracorporeal membrane oxygenation*.

## Discussion

Our study failed to demonstrate clinically significant benefits of simultaneous pulmonary perfusion and ventilation during CPB. These findings are in contrast with Suzuki et al. (13), who reported a significantly shorter duration of postoperative mechanical ventilation and improved alveolar function in infants who were subjected to 30 ml/kg/min pulmonary artery perfusion flows during CPB in the course of corrective surgery for congenital heart disease. Our study was considerably different: all patients were adults, “beating heart” technique rather than hypothermic hyperkalemic cardiac arrest was used whenever possible, and pulmonary perfusion flows were significantly lower (close to 500 ml/min). Clinical studies in settings resembling our study are less sanguine. Selective continuous pulmonary artery perfusion flows of 7 ml/kg/min, comparable to those used in our study, in low-risk adult patients during on-bypass coronary revascularization resulted in no significant clinical benefits (14, 15) despite slightly improved pulmonary perfusion indices and decreased pulmonary tissue inflammatory cytokine production. Importantly, Kiessling et al. (5) reported an absence of clinical benefits despite small trends toward decreased pulmonary tissue-generated inflammatory markers in a study of intermittent selective pulmonary perfusion in high-risk pulmonary patients undergoing on-bypass coronary revascularization. Similar to our results, the latter study demonstrated a statistically insignificant unfavorable trend toward increased overall mortality and pulmonary morbidity. Animal studies of selective partial pulmonary perfusion during CPB reported inconclusive results as well (16), citing a low study size and possibly insufficient duration of the pulmonary artery perfusion.

Studies of alveolar ventilation during CPB suffer from a similar dichotomy between *in vivo* findings and clinical outcomes. In a pig model, Imura et al. (17) demonstrated preservation of transalveolar gas change and metabolic and histologic evidence of protective effects of low-frequency ventilation on histoarchitecture and function of alveolar membrane. However, these protective effects were not clinically evident (6), likely due to large data variability and insufficient study size. In an attempt to increase the power of these studies, a meta-analysis was performed of 16 clinical trials with a total enrollment of 814 patients (7). Various methods of ameliorating iatrogenic atelectasis during CPB included continuous positive alveolar pressure, low-frequency ventilation, and vital capacity recruitment maneuvers. Irrespective of the method, the clinical effects were short lived and did not impact the outcomes.

Outcome studies of fully maintained pulmonary perfusion and ventilation during bypass as in the Drew–Anderson technique (bilateral extracorporeal circulation without a membrane oxygenator, using patients’ lungs as a natural oxygenator) are not without controversy as well. Richter et al. (18) reported a significant decrease in inflammatory cytokines with clinically significant improvements in hemostasis, postoperative pulmonary function, and shorter times of mechanical ventilator support. However, a recent animal study of the same technique (19) did not find any change in inflammatory cytokines production by pulmonary tissue despite electron microscopy evidence of the mitigating effects of pulmonary perfusion and ventilation on polymorphonuclear leukocytes infiltration, interstitial edema formation, and histoarchitecture disruption associated with conventional CPB.

Translating theoretical concepts into clinical outcomes remains an ever-elusive goal. Even such well-established concepts as the deleterious effects of inflammatory cytokines still await their correlation with specific clinical end-points. While Siepe et al. (20) attributed the protective effects of pulmonary perfusion during CPB to the decreased pulmonary tissue expression and blood concentration of inflammatory cytokines, such as IL-6, IL-8, IL-10, and TNF-α, and others (5, 15, 19) could not reproduce these effects. Likewise, despite avoidance of CPB, pulmonary atelectasis and activation of inflammatory cascades associated with pulmonary ischemia–reperfusion injury, off-bypass technique of coronary revascularization offers only a modest clinical impact on pulmonary outcomes in patients with severe pulmonary disease (21–23).

Absence of proof is not a proof of absence. Nevertheless, an apparent lack of clinically significant benefits of pulmonary perfusion and ventilation during CPB begs for an explanation. We could speculate that in a milieu of multiple concurrent operational pathophysiologic mechanisms, mitigating some of the variables may not translate into an observable clinical effect (24). Patients in both groups had comparable incidences of non-modifiable risks of postoperative pulmonary dysfunction and ventilator dependency such as advanced age, reduced preoperative pulmonary function, smoking, renal insufficiency, recent myocardial infarction, and reduced left ventricular function. Untoward effects of CBP may be confounded by altered pulmonary mechanics due to sternothomy, internal mammary artery harvest, pleural entry, and phrenic nerve injury (3, 23, 25–27). In the present study, the majority of the patients in both groups were exposed to blood products perioperatively, an additional and a significant confounding factor associated with a possibility of a delayed lung injury (28). There is also a growing appreciation for the importance of factors not included in our study, such as right ventricular (RV) function and mode of perioperative ventilation. Even though RV performance is not included yet in STS risk score calculation, it had emerged as an important independent factor for perioperative morbidity and mortality in some critically ill populations (29, 30). Similarly, the impact of ventilation strategies on RV pulmonary blood flow functionality and on pulmonary and overall outcomes is increasingly understood and appreciated (31–35). Additionally, the institutional structure, and expertise and experience of individual surgeons, anesthesiologists, and intensivists (23, 36, 37) may be important, but difficult to quantify. While the institutional bias was eliminated in our study, effects of interprovider variability could not be reliably ruled out.

Our study suffers from multiple limitations. It is retrospective and could not be randomized, and strong interprovider variability is likely. A small sample size may have prevented the detection of some significant differences. Some factors, such as RV function and rationale for various decisions and management preferences, were not recorded and their contribution to postoperative outcomes remains unknown. A retrospective differential diagnosis to establish the relative contributions of cardiac and pulmonary pathology in mortality events was difficult in many cases. Nevertheless, we believe the validity of our study, since the largely negative results were obtained despite shorter CPB and cross-clamp times and higher rates of extubation in the OR in the cohort of interest.

In conclusion, our data failed to demonstrate any clinical benefits of simultaneous pulmonary perfusion and ventilation during CPB. It underscores the difficulty to translate attractive theoretical concepts tested in small *in vivo* studies into clinical practice. We believe that multiple confounders and outliers inherent in the care of heterogenous populations may account for our largely negative results. It is eminently possible that the described technique of simultaneous pulmonary perfusion and ventilation during CPB may prove of benefit in highly select groups of patients, such as elderly, those with significant preoperative pulmonary comorbidities, and those with advanced and symptomatic heart failure. As our database is expanded, we hope to be able to differentiate the outcomes of simultaneous pulmonary perfusion and ventilation during CPB according to the type and the severity of preoperative pulmonary comorbidity. Further investigations of the effects of this technique in critically ill patients are needed.

## Author Contributions

YR-B: design, data collection, drafting of the article, and approval of article. AG: concept development, drafting of the article, critical revisions, and approval of article. TS: concept development, critical revisions, and approval of article. KL: data analysis/interpretation, drafting of the article, critical revisions, and approval of article. EG: concept development, design, data analysis/interpretation, drafting of the article, critical revisions, and approval of article.

## Conflict of Interest Statement

The authors declare that the research was conducted in the absence of any commercial or financial relationships that could be construed as a potential conflict of interest.
